# Common Salt in Ague

**Published:** 1852-05

**Authors:** 


*Common Salt in Ague.*—Our readers remember that M. Piorry has for some time been advocating the use of common salt in ague; he has in several instances met with success, as well as M. Scell Montdezert. M. Parant has lately published, in the Transactions of the Medical Society of Toulouse, four cases, in which he gave one ounce of chloride of sodium in six ounces of water, in three doses, two hours before the fit. The cases were all successful; it is, however, not mentioned in how many instances the remedy failed to cure the patients.—*Ibid.*

